# Genome-wide identification of endogenous RNA-directed DNA methylation loci associated with abundant 21-nucleotide siRNAs in *Arabidopsis*

**DOI:** 10.1038/srep36247

**Published:** 2016-10-27

**Authors:** Jian-Hua Zhao, Yuan-Yuan Fang, Cheng-Guo Duan, Rong-Xiang Fang, Shou-Wei Ding, Hui-Shan Guo

**Affiliations:** 1 State Key Laboratory of Plant Genomics, Institute of Microbiology, Chinese Academy of Sciences, Beijing 100101, China; 2 Department of Plant Pathology and Microbiology, Institute for Integrative Genome Biology, University of California, Riverside, CA 92521, USA

## Abstract

In *Arabidopsis*, the 24-nucleotide (nt) small interfering RNAs (siRNAs) mediates RNA-directed DNA methylation (RdDM) and transcriptional gene silencing (TGS) of transposable elements (TEs). In the present study, we examined genome-wide changes in DNA methylation and siRNA accumulation in *Arabidopsis* induced by expression of the *Cucumber mosaic virus* silencing suppressor protein 2b known to directly bind to both the 21/24-nt siRNAs as well as their associated Argonaute proteins. We demonstrated a genome-wide reduction of CHH and CHG methylation in the 2b-transgenic plants. We found that 2b suppressed RdDM not only at the previously annotated loci directed by 24-nt siRNAs but also a new set of loci associated with 21/22-nt siRNAs. Further analysis showed that the reduced methylation of TEs and coding genes targeted by 21/22-nt siRNAs was associated with sequestration of the duplex siRNAs by the 2b protein but not with changes in either siRNA production or transcription. Notably, we detected both the deletion and/or the transposition of multicopy TEs associated with 2b-induced hypomethylation, suggesting potential TE reactivation. We propose that the silencing of many TEs in *Arabidopsis* is controlled by the 24- and 21-nt endogenous siRNAs analogous to *Drosophila* TE silencing by PIWI-interacting RNAs and siRNAs.

The methylation of cytosines in nuclear DNA is a conserved epigenetic silencing mechanism and controls many important biological processes, including defense against transposon proliferation, control of genomic imprinting, and the regulation of gene expression, which imparts an additional layer of heritable information upon the DNA code^1^,^2^,^3^,^4^. In plants, three main DNA methylation pathways mediate the methylation of CG, CHG, and CHH (where H = A/T/C) sequence contexts. METHYLTRANSFERASE 1 (MET1) is responsible for the maintenance of CG methylation, and CHG methylation is maintained by CHROMOMETHYLASE 3 (CMT3). DOMAINS REARRANGED METHYLTRANSFERASES 1 (DRM1) and 2 (DRM2) are responsible for CHH methylation through the RNA-directed DNA methylation (RdDM) pathway^5^,^6^. RdDM triggered by 24-nucleotide (24-nt) small interfering RNAs (siRNAs) has been studied extensively^7^,^8^. Although recent data demonstrated that DNA methylation is not always associated with the accumulation of corresponding siRNAs, transgenerational maitenance of gene body CHG and CHH methylation required RdDM^9^,^10^,^11^.

In plants, the RdDM pathway involves a plant-specific RNA polymerase, Pol IV, which transcribes hetero-chromatic regions into non-coding transcripts. These transcripts are converted into double-stranded (ds)RNA precursors by RNA-dependent RNA polymerase 2 (RDR2). Dicer-like enzyme 3 (DCL3) acts on these precursors and processes them into 24-nt siRNAs. The resulting 24-nt siRNAs are loaded into ARGONAUTE 4 (AGO4), AGO6, or AGO9 complexes to target Pol V-dependent nascent scaffold transcripts and recruit the DRM2 to guide *de novo* cytosine DNA methylation and maintain transcriptional gene silencing (TGS) at Pol V-transcribed loci^12^,^13^.

In addition to the canonical RdDM mediated by 24-nt siRNAs (herein referred to as 24-nt-siRdDM), several recent studies support a role for 21-22-nt siRNAs in directing DNA methylation and maintenance of the silencing of a few selected loci. Several silencing-related factors that have been previously implicated only in post-transcriptional gene silencing (PTGS), including RDR1, RDR6, AGO1, AGO2, and DCL2, are involved in 21-22-nt siRNA-mediated RdDM of some transposable elements (TEs) and several intergenic regions^14^,^15^. Recently, 15 TE subfamilies with the individual loci undefined, were reported to accumulate RDR6-dependent 21-22-nt siRNAs in a *ddm1* mutant background^15^. In addition to the TEs, two endogenous *TAS* loci (trans-acting small interfering RNAs, ta-siRNAs) also display a high cytosine methylation status at ta-siRNA-generating regions, and 21-22-nt ta-siRNAs are required to guide the DNA methylation of *TAS* loci^14^.

Comprehensive methylome analyses of many *Arabidopsis* silencing mutants have shown that among all of the mutants that are not involved in canonical 24-nt-siRdDM, the *rdr1* mutant was even stronger than the *rdr6* mutant in reducing the methylation of all CG, CHG, and CHH contexts in chromosome 1^16^ in regions that were more likely to be associated with genes and 21-22-nt siRNAs in wild-type *Arabidopsis*^17^. These data suggest that proteins other than RDR6, such as RDR1, that generate 21-22-nt siRNAs play a role and affect more loci in DNA methylation. Thus, genome-wide 21-22-nt siRNA-related methylation loci (herein referred to as 21/22-nt-siRdDM to distinguish the canonical 24-nt-siRdDM) remain to be elucidated.

Plant DNA viruses encode viral suppressors of RNA silencing (VSRs) that interfere with DNA methylation by mainly disrupting the methylcycle. For example, geminiviral VSRs such as AC2/AL2, C2/L2 and βC1 act by inhibiting the adenosine kinase (ADK) activity that plays a crucial role in adenosine and methylcycle maintenance or cytokinin regulation^18^,^19^,^20^, by interacting with S-adenosylmethionine decarboxylase 1 (SAMDC1), which catalyzes the conversion of S-adenosylmethionine (SAM) to decarboxylated SAM (dcSAM)^21^, or by inhibiting the activity of S-adenosyl-homocysteine-hydrolase (SAHH), which is also involved in the methylcycle^22^. Recently studies reported that potyviral helper component proteinase (HCPro) and Crinivirus coat protein also suppress antiviral RNA silencing through disruption of the methylcycle^23^,^24^. In contrast, VSR protein 2b encoded by the positive-strand RNA virus *Cucumber mosaic virus* (CMV) suppresses the DNA methylation associated with PTGS^25^,^26^ and binds to duplex siRNAs in 21-, 22- and 24-nt classes as well as AGO proteins^27^,^28^,^29^. The suppression of PTGS and DNA methylation in a few selected 24-nt-siRdDM loci (e.g. *MEA-ISR* and *SN1*) by CMV 2b protein requires siRNA-binding activity but is independent of its ability to interact directly with AGOs^28^. However, the 2b-AGO interaction may facilitate the 2b suppression function because 2b-AGO interactions in the nucleolus redistribute both the 2b and AGO proteins in the nucleus^28^ and inhibit AGO1 slicer activity in *vivo*^30^. However, it remains unknown if 2b binds to siRNAs *in vivo* and interferes with genome-wide DNA methylation.

In the present study, we discovered a genome-wide reduction of CHH and CHG methylation in 2b-transgenic *Arabidopsis* plants (line 2b-3) expressing the 2b protein encoded by the severe Shan-Dong (SD) isolate from CMV sub-group I. We also found that the 2b protein co-immunoprecipitated 21-, 22- and 24-nt duplex siRNAs *in vivo*, with a preference for 21- and 22-nt siRNAs. By characterizing the differential methylation regions (DMRs) associated the total and 2b-bound siRNAs, we discovered a subset of endogenous 21/22-nt-siRdDM loci, including TEs and coding genes, in both gene bodies and upstream regions. The transcriptome analysis revealed that the DNA methylation status at most 21/22-nt-siRdDM loci did not impact their expression. However, DNA blotting hybridization detected deletion and/or transposition of multicopy TEs with reduced methylation in the presence of the 2b protein, suggesting potential reactivation of multicopy TEs by the 2b protein.

## Results

### Identification of differential methylation regions (DMRs) between wild type and 2b transgenic plants

**Figure 1:**
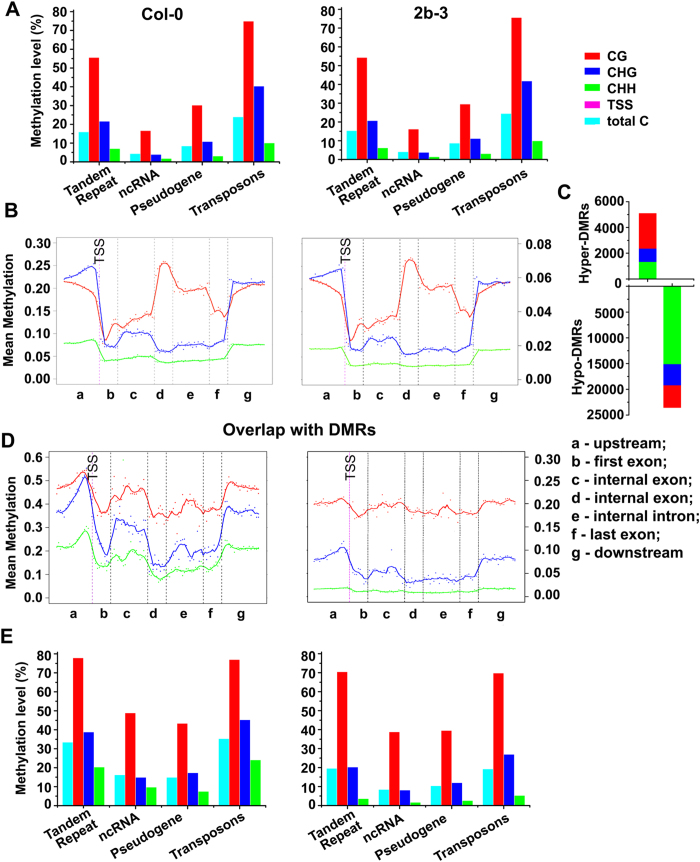
Bisulfite sequencing profiling of methylation in wild type Col-0 and 2b transgenic plants. (**A**) The average genome-wide methylation level of tandem repeats, ncRNA, pseudogenes and transposons. (**B**) The average methylation level of coding genes genome-wide. The mean methylation level of CG (red line) refers to the y-axis on the left, and that of CHG and CHH (blue and green lines) refers to the y-axis on the right. (**C**) Comparison of the levels of hyper- and hypo-DMRs in CG, CHG and CHH cytosine contexts between Col-0 and 2b-3 plants. (**D**) The average methylation level of coding genes in DMRs. (**E**) The average methylation level of tandem repeats, ncRNA, pseudogenes and transposons in DMRs.

The 2b-transgenic *Arabidopsis* plants created by transformation of the full-length 2b coding sequence from the severe Shan-Dong (SD)CMV isolate^28^ was used in this study. The accumulation of the 2b protein in 2b-transgenic line 2b-3 was confirmed (Figure S1A). We first compared genome-wide DNA methylation patterns in Col-0 and 2b-3 by bisulfite sequencing. We found that tandem repeat, non-coding (nc)RNA, pseudogenes and transposons were highly methylated in both Col-0 and 2b-3 plants (Fig. 1A). The DNA methylation levels in CG, CHG and CHH of the expressed genes showed a tendency to decrease at transcription start sites (TSS), but the methylation level of CG to increase at gene bodies (Fig. 1B), which was consistent with previous reports for the whole genome methylation sequencing results in Col-0^31^,^32^ and 2b-3 (Figures S1 and S2). Next, we identified sequences that were differentially methylated in Col-0 and 2b-3 by calculating methylation levels in the context of each cytosine and defined DMRs in 2b-3 plants in which the DNA methylation levels varied significantly compared with Col-0 plants. This led to the identification of 23,552 hypomethylated DMRs (hypo-DMRs) and 5,093 hypermethylated DMRs (hyper-DMRs) (Fig. 1C), which made up 3.31% of the genomic sequence in total. Separation into each cytosine context revealed a high proportion of CG in hyper-DMRs (2,729/5,093) compared with hypo-DMRs (4,298/23,552); however, a remarkable amount (15,141) of CHH in hypo-DMRs was obtained (Fig. 1C), in agreement with the observation of a subtle genome-wide decrease in CHH content in 2b-3 compared with Col-0 (Fig. 1B) (P > 0.05, t-test).

### Decreased CHH/CHG methylation of genomic sequences in 2b transgenic plants

The identified DMRs were located not only in coding genes but also in tandem repeats, ncRNA, pseudogenes and TEs (Fig. 1D,E). For the coding genes, while the average methylation in the CG context was slightly affected by 2b, the average methylation of CHG and especially of CHH was greatly impaired either in the gene body or upstream and downstream of genes in 2b-3 (Fig. 1D). Note that the number on the y-axis for DMR coding genes was 0.6 and 0.3 for CG and CHG/CHH, respectively (Fig. 1D), but the number for coding genes in the whole genome was 0.3 and 0.08 for CG and CHG/CHH, respectively (Fig. 1B), showing that the average methylation level for coding region DMRs was much higher than that of the total coding genes in the whole genome. These data suggested that coding genes with a high density of methylation were targeted by the 2b protein, leading to the decreased CHH and CHG methylation observed in 2b-3 plants (Fig. 1D). A high density of methylation was also detected for DMRs located in tandem repeats, ncRNA, pseudogenes and TEs compared to that in the whole genome (compare Fig. 1A,E). Again, the average methylation level for the DMRs in those loci was also greatly reduced, especially in CHH, in 2b-3 (Fig. 1E). These data together indicated that stable expression of the 2b protein reduced genome-wide CHH and CHG methylation in *Arabidopsis* plants, providing a distinct in *vivo* model to investigate the mechanism of the RdDM^6^,^28^.

### Identification and characterization of small RNAs in 2b transgenic plants

The 2b protein has been shown to be capable of binding to 21-, 22-, and 24-nt siRNAs^28^. Therefore, we investigated the profiles of siRNAs in Col-0 and 2b-3 plants by sequencing the total small RNAs from Col-0 and 2b-3 plants, as well as those co-immunoprecipitated with the 2b protein from 2b-3 plants (Table S1).

**Figure 2:**
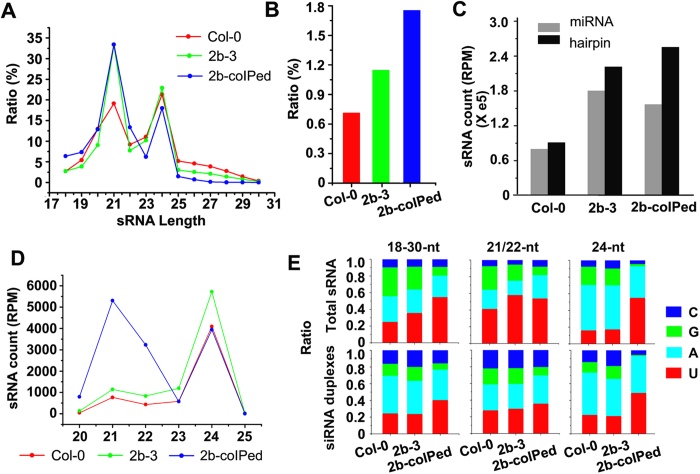
Deep sequencing profiling of small RNAs in wild type Col-0 and 2b transgenic plants. (**A**) Proportions of different lengths of total siRNAs from Col-0, 2b-3 plants and 2b-coIPed complexes. (**B**) The ratio of perfectly matched siRNA duplexes in each siRNA library. (**C**) The numbers of miRNAs and potential miRNA/miRNA* duplexes (labeled as hairpins) by small RNAs that matched with the annotated miRNA precursor sequences in each siRNA library. (**D**) The proportion of each length of perfectly paired siRNA duplex. (**E**) The 5′ terminal nucleotide compositions of total siRNA and siRNA duplexes in different libraries (left columns for total siRNAs,middle for 21/22-nt and right for 24-nt siRNAs).

Consistent with a previous study^33^, total siRNAs extracted from Col-0 were dominated by 21- and 24-nt species, with a slightly greater population of 24-nt siRNAs compared with 21-nt siRNAs (Fig. 2A). However, 21-nt siRNAs became much more abundant in 2b-3 plants, while the levels of 24-nt siRNAs remained comparable to those in Col-0 plants (P < 0.001, proportion test) (Fig. 2A). The endogenous siRNAs co-immunoprecipitated with the 2b protein from 2b-3 plants (referred to as 2b-coIPed siRNAs) was similar in the length distribution to the total siRNAs of 2b-3 plants sequenced without co-immunoprecipitation (Fig. 2A). Indeed, perfectly matched siRNA duplexes represented 0.71% of the Col-0 siRNA library, but increased to 1.15% and 1.75%, respectively, in the 2b-3 and 2b-coIPed siRNA libraries (Fig. 2B). In addition to the perfect duplexes, the 2b protein also exhibited a high affinity for imperfect siRNA duplexes that had formed between micro(mi)RNA/miRNA*^28^. The miRNAs* were scoped by matching siRNAs to miRNA precursor hairpin sequences, and the levels of miRNAs* increased in 2b-3 (Fig. 2C), which may account for the stabilization and increased levels of miRNAs in 2b-3 plants. A large amount of miRNAs and miRNAs* in the 2b-coIPed siRNA library suggested that the miRNA/miRNAs* duplexes were likely co-precipitated by the 2b protein (Fig. 2C). Previous *in vitro* reciprocal-competing binding assays showed that 2b possessed a similar binding affinity for the 21- and 24-nt siRNA duplexes^28^. Thus, the ratio of each length of perfectly paired siRNA duplex (not including the miRNA/miRNA* duplexes) was counted. Despite a large amount of 24-nt duplexes in Col-0 and 2b-3 plants, the 21-nt siRNA duplexes were markedly increased in the 2b-coIPed siRNA library (P < 0.001, chi-square test) (Fig. 2D). This finding suggests an *in vivo* preference for the 2b protein to bind 21-nt siRNA duplexes, which may explain why 21-nt siRNAs were more abundant in 2b-3 plants than Col-0 plants.

Next, we assessed the features of the 2b-coIPed siRNAs by comparing the 5′-terminal nucleotides of the siRNA duplexes in the Col-0, 2b-3 and 2b-coIPed libraries. Figure 2E shows that, while the proportion of the siRNAs duplexes with 5′-terminal adenine (1A) from 2b-3 plants both before (39.7%) and after 2b co-immunoprecipitation (36.5%) was slightly lower than that in Col-0 (45.0%) (Fig. 2E), the proportion of 1U siRNA duplexes was markedly increased in the 2b-coIPed siRNAs, especially for 24-nt, compared with the Col-0 and 2b-3 siRNA duplexes (Fig. 2E) (P < 0.001, proportion test). The previous studies showed that the *in vitro* 2b binding siRNA assay had no obvious binding preference for siRNAs beginning with different nucleotides at the 5′ terminus^28^, and that the 2b protein potentially interacted directly with the AGO1 and AGO4 proteins^27^,^28^,^29^. It is known that AGO1 mainly binds to 21-nt 1U miRNAs and siRNAs, but also approximately 30% of 24-nt 1U siRNAs species and that 2b interacts with AGO1 in *vivo*^3^,^28^,^33^,^34^,^35^. Thus, our data together suggest that the binding of the 2b protein directly to siRNA duplexes and indirectly to siRNAs in AGO1 complex may contribute to the population structure of the endogenous siRNAs found in 2b-3 plants.

### Correlation between DMRs and siRNAs in wild type and 2b transgenic plants

**Figure 3:**
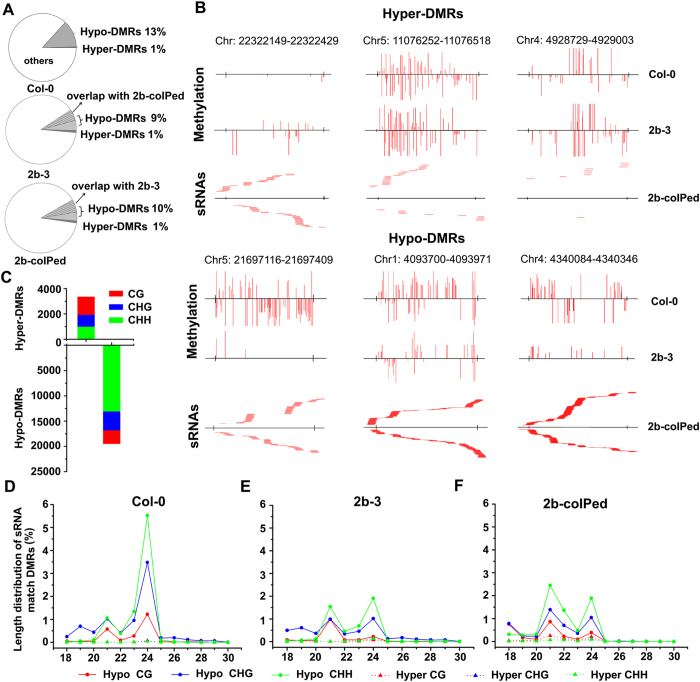
Analysis of the correlation between DMRs and siRNAs in wild type Col-0 and 2b transgenic plants. (**A**) The ratio of siRNAs that matched with hyper- and hypo-DMRs from the Col-0, 2b-3 and 2b-coIPed siRNA libraries. Slash populated areas mark the overlap siRNAs between 2b-3 and 2b-coIPed siRNA libraries. (**B**) Examples of hyper- and hypo-DMRs corresponding to 2b-coIPed siRNAs in different chromosome regions. Vertical lines show the methylation level of each cytosine in Col-0 and 2b-3. Horizontal lines show the 2b-coIPed siRNAs that matched with the DMRs. (**C**) The numbers of 2b-coIPed siRNAs that matched with the hyper- or hypo-DMRs in CG, CHG and CHH contexts. (**D–F**) Proportion of different lengths of siRNAs from each siRNA library that matched with the hypo-DMRs (solid lines) and hyper-DMRs (dotted lines) in CG, CHG and CHH contexts.

Next, we analyzed the correlation between the identified DMRs and the siRNAs in the three libraries. 13.28% of the total siRNAs found in Col-0 were mapped to the identified DMRs, with 13% localized to the hypo-DMRs and the remaining 0.28% to the hyper-DMRs. 10% and 11% of the total and 2b-coIPed siRNAs from 2b-3 plants were mapped to the identified DMRs, and again, mostly to hypo-DMRs in both cases, and more than 50% of DMR-associated 2b-coIPed siRNAs overlapped with DMR-associated siRNAs from 2b-3 plants (Fig. 3A). When these siRNA-targeted DMRs were divided into the three context sequences, it became apparent that the DMR-associated siRNAs found in 2b-coIPed siRNA library were preferentially mapped to CHH hypo-DMRs (Fig. 3C, Table S2). Figure 3B shows examples of hyper- and hypo-DMRs corresponding to 2b-coIPed siRNAs in different chromosome regions. We further examined the relative abundance of the DMR-associated siRNAs in different sizes mapped to hypo- and hyper-DMRs in the three context sequences (Fig. 3D–F). We found that a high density of siRNAs aggregated with hypo-DMRs, especially with CHG and CHH in hypo-DMRs (Fig. 3D–F, solid lines), whereas a very small portion of the siRNAs in the three libraries were mapped to hyper-DMRs (Fig. 3D–F, dashed lines). As shown in Fig. 3D, a high proportion of 24-nt siRNAs from Col-0 plants were mapped to CHG and CHH hypo-DMRs, consistent with the known role of the 24-nt siRNAs involved in canonical RdDM processes^7^,^8^. The DMR-associated siRNAs in both the total and 2b-coIPed siRNA libraries from 2b-3 plants also aggregated with CHG and CHH hypo-DMRs (Fig. 3E,F); however, the relative abundance of the DMR-associated 24-nt siRNAs in both libraries was significantly reduced compared to that from Col-0 plants (P < 0.001, proportion test). These findings indicate that expression of the 2b protein in *Arabidopsis* plants specifically reduced the levels of the 24-nt siRNAs associated with the identified hypo-DMRs (Fig. 3D–F) without altering the accumulation of the total 24-nt siRNAs (Fig. 2A).

By comparison, the accumulation of the 21-nt siRNAs associated with the identified hypo-DMRs in 2b-3 plants was similar to that in Col-0 plants (Fig. 3E) unlike the total 21-nt siRNAs, which were much more abundant in 2b-3 plants than Col-0 plants (Fig. 2A). However, we detected a modest enrichment of these hypo-DMRs-associated 21-nt siRNAs in the 2b-coIPed siRNAs library (Fig. 3F), suggesting *in vivo* sequestration of the DMR-associated 21-nt siRNAs by the 2b protein. Therefore, it is likely that both the reduced production of the DMR-associated 24-nt siRNAs and the sequestration of the DMR-associated 21-nt siRNAs play a role in the genome-wide reduced DNA methylation detected in 2b-3 plants.

### Categorization of hypo-DMR loci with various lengths of 2b-coIPed siRNAs

To examine the hypothesis, we divided the identified hypo-DMR loci into five groups according to the relative abundance of the DMR-associated 24-nt and 21-/22-nt siRNAs in the 2b-coIPed siRNA library. The hypo-DMR loci in group I and V were associated only with 24-nt siRNAs (marked as 2b-24^only^) and 21-22-nt siRNAs (2b-21/22^only^), respectively. Group II hypo-DMRs were targeted by 24-nt siRNAs that were at least two times in excess of the 21- and 22-nt siRNAs (2b-24^mainly^) whereas group IV hypo-DMRs were targeted by 21- and 22-nt siRNAs that were at least two times in excess of the 24-nt siRNAs (2b-21/22^mainly^). Hypo-DMR loci that were associated with an equivalent amount of 24-nt and 21-22-nt siRNAs were included in group III (2b-24^≈21/22^).

**Figure 4:**
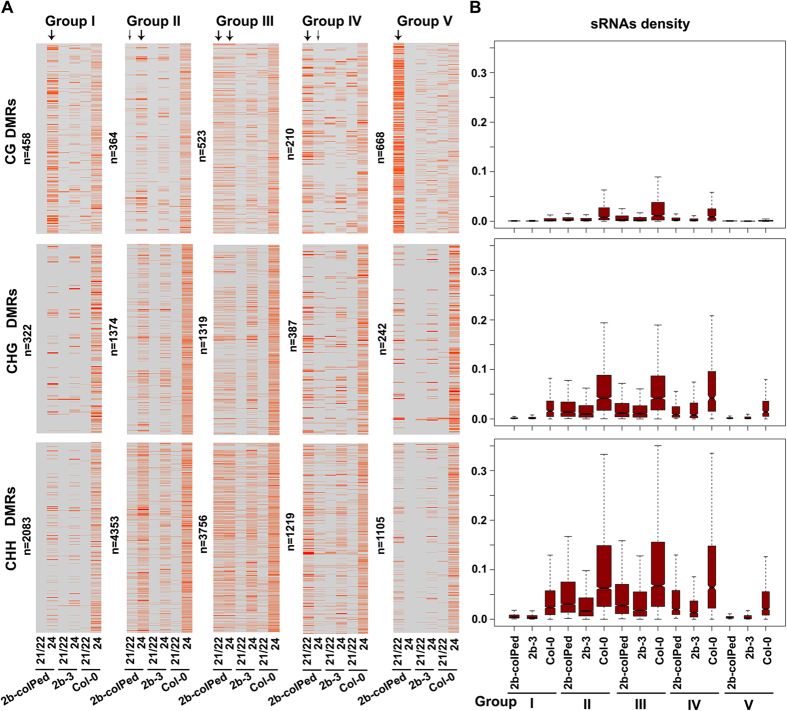
Categorization of hypo-DMR loci with various lengths of 2b-coIPed siRNAs. (**A**) Heatmap of the siRNA density within each hypo-DMR in CG, CHG and CHH contexts. Columns represent data for 21/22- and 24-nt siRNAs from Col-0, 2b-3 and 2b-coIPed siRNA libraries that matched to the five groups of hypo-DMR loci. The numbers of hypo-DMR loci in each group are shown (n). The arrows mark the siRNAs in the 2b-coIPed library. The thickness of the arrows represents the abundance of 2b-coIPed siRNAs. The density of matched siRNAs to each hypo-DMR locus (row) coincided with the degree in red for each individual heatmap, but they are not comparable among each other. (**B**) Box plots of the siRNA density for each group of hypo-DMRs in Col-0, 2b-3 and 2b-coIPed siRNA libraries.

2,863 of the 18,383 hypo-DMR loci (15.57%) were associated only with 24-nt siRNAs (2b-24^only^), and included 458 CG loci, 322 CHG loci and 2083 CHH loci. As expected, mainly 24-nt 1A siRNAs from Col-0 plants were mapped to these loci (Fig. 4A and S3), despite the overall low density of siRNAs in Col-0 (Fig. 4B). The levels of DMR-associated 24-nt siRNAs were greatly reduced in 2b-3 plants (P < 0.001, Mann-Whitney U test) (Fig. 4A), indicating that group I loci corresponded to those loci targeted by the canonical RdDM mediated by AGO4-loaded 24-nt siRNAs^13^.

63.59% of the identified hypo-DMR loci belonged to groups II and III (Fig. 4A). Group II hypo-DMRs (2b-24^mainly^) included 364 for CG, 1374 for CHG and 4353 for CHH while those in group III (2b-24^≈21/22^) contained 523 for CG, 1319 for CHG and 3756 for CHH (Fig. 4A), indicating enrichment for CHH (69.37%) and CHG (23.04%) hypo-DMRs. Loci in these two groups were associated with a high density of siRNAs in Col-0 plants (Fig. 4B). There was a large amount of 24-nt 1A siRNAs mapped to hypo-DMRs, especially CHH, in Col-0 plants, and the amount was significantly reduced in 2b-3 plants (P < 0.001, Mann-Whitney U test) (Fig. 4A and S3), suggesting that hypomethylation in the loci of groups II and III resulted from the reduced production of the 24-nt siRNAs in 2b-3 plants. However, the involvement of the 21-22-nt siRNAs in RdDM at these loci cannot be excluded since their sequestration in the 2b complex.

2b-21/22^mainly^ (group IV) contained 1,816 hypo-DMR loci (9.88%), which included 210 loci for CG, 387 (21.3%) for CHG and 1,219 (67.1%) for CHH (Fig. 4A). Group IV loci were associated with a high density of siRNAs and a large amount of 24-nt 1A siRNAs in Col-0 plants (Fig. 4B and S3), suggesting that they play a role in RdDM at these loci. A reduction of the 24-nt siRNAs in 2b-3 plants might account for the hypomethylation at these loci; however, because the majority of the 2b-coIPed siRNAs were 21-22 nt in length, we also propose a role for 21-22-nt siRNAs in RdDM at these loci in Col-0 plants (see below section). Indeed, 2b-21/22^mainly^ included the *TAS1C* and *TAS3* loci, in which DNA methylation has been found to be directly guided by 21-22-nt siRNAs despite the presence of existing 24-nt tasiRNAs at these loci^14^.

2,015 of the 18,383 hypo-DMR loci (10.96%) was associated only with 21- and 22-nt siRNAs and these group V loci included 668 for CG, 242 for CHG and 1105 for CHH (Fig. 4A). The density of siRNAs in these loci was low (Fig. 4B), but notably, the levels of 24-nt siRNAs was still much higher compared with the 21-22-nt siRNAs in Col-0 plants and were greatly reduced in 2b-3 plants, especially with respect to the CHG and CHH hypo-DMRs (P < 0.001, Mann-Whitney U test) (Fig. 4A). The majority of the 1A 24-nt siRNAs at these loci in Col-0 (Figure S3) were suggested to a play role in RdDM. However, despite potential interference with RdDM by the interaction with 2b-AGO4, 2b-coIPed only 21-22-nt siRNAs at these hypo-DMR loci, and the decreases in 24-nt siRNAs in 2b-3 indicated that, like the above hypo-DMR group IV, the 21-22-nt siRNAs probably played a role in RdDM at these endogenous loci.

Taken together, our data for the hypo-DMRs from group IV and V suggest that 21-22-nt siRNAs participate, by either directly guiding or alongside 24-nt siRNAs, in DNA methylation at many endogenous loci.

### Identification of hundreds of TE loci targeted by highly abundant 21- and 22-nt siRNAs

**Figure 5:**
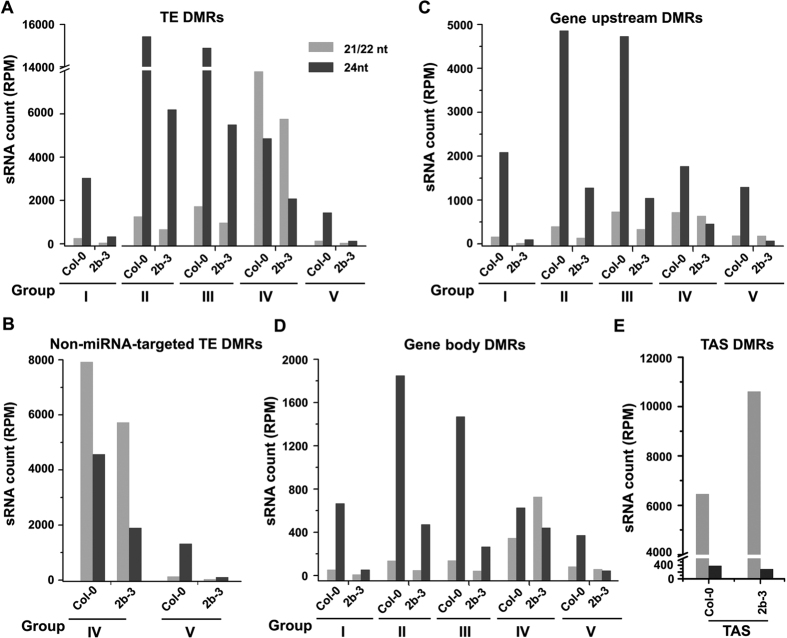
Numbers of various lengths of siRNAs that matched with TEs and coding genes in each hypo-DMR group. (**A,B**) The numbers of siRNAs that matched with all TE loci (**A**) or only non-miRNA-target TE loci (**B**) in the indicated hypo-DMR groups. (**C,D**) The numbers of siRNAs that matched with the hypo-DMRs located in upstream regions of coding genes (**C**) and gene bodies (**D**) in five hypo-DMR groups. (**E**) The numbers of siRNAs that matched with the *TAS* genes in hypo-DMR group IV.

A total of 8468 individual TE elements from all 18 superfamilies (including all 318 subfamilies) contained the identified hypo-DMRs (Figure S4), which is consistent with TEs as major targets of RdDM^36^. Most (79.7%) of these TEs were targeted by the hypo-DMRs in groups I, II and III; however, the hypo-DMRs in groups IV and V were identified in a large number (1719) of the targeted TE elements (Figure S4). We next calculated the total number of the 24-nt siRNAs and 21-/22-nt siRNAs from Col-0 and 2b-3 plants mapped to the TEs in each of the five groups (Fig. 5A). High levels of 24-nt siRNAs were mapped to the TEs in each of the five groups in Col-0 plants and their accumulation was greatly reduced in 2b-3 plants (Fig. 5A), supporting a major role for 24-nt siRNAs to target TEs for RdDM.

Notably, we found that the 21-/22-nt siRNAs mapped to the TEs with group IV hypo-DMRs of either Col-0 or 2b-3 plants were distinctly much more abundant than both the 24-nt siRNAs mapped to the same group of TEs and the 21-/22-nt siRNAs mapped to the TEs of the remaining four groups (Fig. 5A). This group contained 960 TEs in total from each of the 18 superfamilies (Figure S4). Recently studies reported that miRNAs-directed secondary 21/22-nt siRNAs biogenesis from many TEs when they are epigenetically reactivated (named as easiRNAs)^37^,^38^. To rule out the group IV possible contains miRNAs-targeted TEs, we analyzed all known miRNA-targeted TEs in *Arabidopsis* and found the group IV contained 139 loci in miRNA-targeted TEs^38^. However, more than 90% of the 21-/22-nt siRNAs mapped to the group IV TEs remained highly abundant (P < 0.001, chi-square test) after removing 21-/22-nt siRNAs mapped to the miRNA-targeted TE loci (Fig. 5B).

We further examined the 15 TE subfamilies identified previously as potential targets of RdDM by RDR6-dependent 21-22-nt siRNAs^15^. We found that 829 TE elements in the 15 subfamilies from superfamilies *LTR/Copia*, *RC/Helitron*, *LTR/Gypsy* and *DNA/En-Spm* contained hypo-DMRs in groups IV and V. However, most (3267 loci) of the TEs in the 15 subfamilies were targeted by hypo-DMRs in groups I, II and III (Figure S4), and were associated with highly abundant 24-nt siRNAs (Fig. 5A). Thus, it is unlikely that RdDM targeting all of the TEs in these 15 subfamilies relied on 21-22-nt siRNAs.

### Identification of a subset of coding genes targeted by highly abundant 21-22-nt siRNAs

Fewer hypo-DMRs (4483 loci) from the five groups were localized to the coding genes compared to the hypo-DMRs specific to TEs, including 2867 loci at upstream promoters and 1616 loci in coding sequence regions (CDS). Mapping of the total 24-nt siRNAs and 21-/22-nt siRNAs from Col-0 and 2b-3 plants to the upstream promoters and CDS in each of the five groups revealed shared features with the siRNAs associated TEs (Fig. 5C,D). The levels of 24-nt siRNAs mapped to the coding genes in each group in Col-0 were greatly reduced in 2b-3 (P < 0.001, chi-square test) (Fig. 5D,E). We also found that the 21-/22-nt siRNAs mapped to 172 group IV CDS loci were more abundant than the 21-/22-nt siRNAs mapped to the CDS loci of the remaining four groups in both Col-0 and 2b-3 plants (Fig. 5D), suggesting possible involvement of 21-/22-nt siRNAs in regulating the methylation of these CDS loci. Interestingly, we detected an increased accumulation of the 21-/22-nt siRNAs mapped to this group of CDS loci in 2b-3 plants than Col-0 plants, in particular for 5 of the 7 *Arabidopsis TAS* loci identified in this group (Fig. 5E). In Col-0 plants, *TAS3B* and *TAS4* contained no hypo-DMRs and exhibited very low levels of DNA methylation or siRNAs (Figure S5). In contrast, *TAS1A*, *TAS1B*, *TAS1C*, *TAS2*, and *TAS3* were heavily methylated and targeted by abundant siRNAs predominantly 21- and 22-nt in length in Col-0 plants. The hypomethylation of the five *TAS* loci in 2b-3 plants was associated with the strong sequestration of the 21-/22-nt siRNAs mapped to the same loci (Figure S5), indicating a role for *TAS*-specific 21-22-nt siRNAs in the DNA methylation of *TAS* loci as reported previously for *TAS1C* and *TAS3*^14^.

### The involvement of 21-22-nt siRNAs in RdDM does not impact the expression of the target loci

We next compared the transcription levels of the coding genes containing hypo-DMRs in groups IV and V in Col-0 and 2b-3 plants by transcriptome sequencing. 126 of the 172 CDS loci in group IV were detected in either Col-0 or 2b-3 plants and the expression levels of these genes were generally low. Only 11 of the 126 CDS loci and 27 of the 665 promoter loci in group IV showed a significant increase in the transcription in 2b-3 (P < 0.05, FDR < 0.001) (Figure S6A,B) and the expression levels of the five *TAS* genes did not exhibit significant differences between Col-0 and 2b-3 plants (Figure S6C, green dots). Similarly, only 26 of the 521 CDS loci and 30 of the 601 promoter loci in group V displayed a significant increase in transcription in 2b-3 plants compared with Col-0 plants (P < 0.05, FDR < 0.001) (Figure S6D,E). Taken together, our data suggest that neither DNA methylation nor the sequestration of the 21-/22-nt siRNAs by the 2b protein has an obvious effect on the transcription of the majority coding genes in groups IV and V, consistent the earlier observations^14^.

### 2b-mediated hypomethylation destabilizes multicopy numbers of TE loci

**Figure 6:**
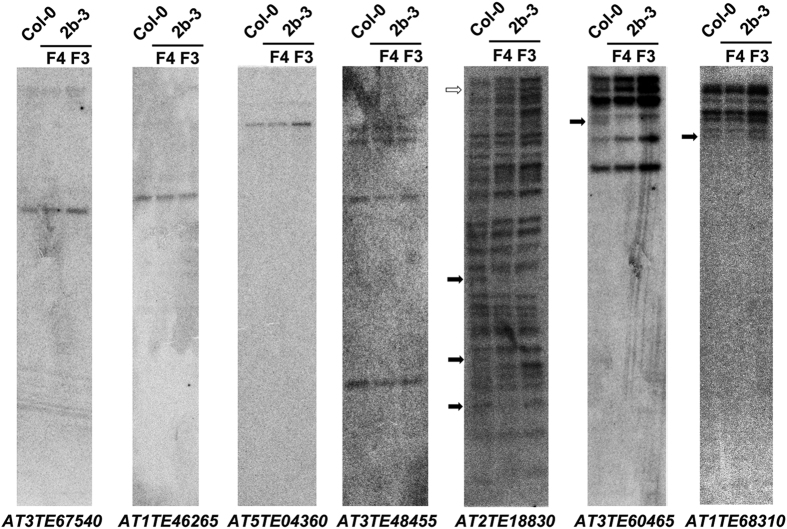
Detection of TE transposition in the third and fourth generations of 2b transgenic plants. DNA blotting analysis of seven TEs that overlapped with hypo-DMRs. The DNA samples were extracted from wild type Col-0, and the third (F3) and fourth (F4) generations of 2b-3 plants. Hollow arrow indicate additional hybridization bands in F3 and F4 compared with Col-0; solid arrows indicate the lack of a hybridization band in (F3) and/or (F4) compared with Col-0.

To evaluate the impact of the hypomethylation of TEs induced by the 2b protein, Southern blots were performed to examine the copy number of several TEs containing hypo-DMRs associated with siRNAs co-immunoprecipitated with the 2b protein. These included four loci in the superfamily *LTR/Gypsy (AT2TE18830*, *AT3TE60465*, *AT3TE67540*, and *AT1TE46265*, which were classified in the *ATLANTYS1*, *ATLANTYS2*, *ATGP3* and *ATHILA6B* families, respectively), two loci in the *DNA/MuDR* superfamily (*AT1TE68310* and *AT5TE04360* in the *VANDAL2N1* and *ATMU10* families, respectively), and one locus, *AT3TE48455*, in the *LINE/L1* superfamily. As showed in Fig. 6, three TEs with one hybridization band in Col-0 (*AT3TE67540*, *AT1TE46265* and *AT5TE04360*) remained unaltered in the third generation (F3) and F4 of the 2b-3 plants. Four hybridization bands of *AT3TE48455* also remained unchanged in the 2b-3 plants. However, loss of one or more hybridization bands was observed for the TEs (*AT2TE18830*, *AT3TE60645*, and *AT1TE68310*) with high copy numbers in F4 of the 2b-3 plants compared with Col-0 (Fig. 6). A clear lack of one hybridization band was detected for *AT3E60465* (group IV) in both F3 and F4 of 2b-3 plants and for *AT1TE68310* (group II) in F4 but not in F3 of 2b-3 plants, and the absence of at least three hybridization bands was detected for *AT2TE18830* (group IV) in F4 with two of them absent in F3 of 2b-3 plants (Fig. 6). We also detected one additional band for *AT2TE18830* in both F3 and F4 of 2b-3 plants (Fig. 6). The possibility of an incomplete restriction enzyme digestion was ruled out because three biological replicates were performed and similar results were obtained. These results suggested that 2b-mediated hypomethylation caused the destabilization of TEs, resulting in potential TE deletion and/or transposition in 2b-3 transgenic plants.

## Discussion

The CMV silencing suppressor 2b protein exhibits complex biochemical and subcellular targeting activities, including the suppression of RNA silencing in the cytoplasm and DNA methylation in the nucleus. Our bisulfite sequencing analysis result revealed a genome-wide reduction of CHG and, especially, CHH methylation in 2b-transgenic *Arabidopsis* plants, and identified the DMRs between wild-type and 2b-transgenic plants, consistent with the previous notion that the 2b protein plays a role in the suppression of RdDM required for constant *de novo* CHH methylation^6^.

Most of the previous researches investigating RdDM have constructed a canonical RdDM that *de novo* DNA methylation is mediated by 24-nt siRNAs (24-nt-siRdDM). In the present study, aided by the capability of the 2b protein to bind both the 21-22-nt and 24-nt ds-siRNAs as well as their associated AGO proteins and suppress DNA methylation^28^, we was able to characterize DMRs associated the total and 2b-bound siRNAs. We found that 2b suppressed DNA methylation not only at the previously annotated 24-nt-siRdDM loci but also a new set of endogenous 21/22-nt-siRdDM loci, including TEs and coding genes, in both gene bodies and upstream regions.

We found that a large amount of 21-22-nt siRNAs were matched with the endogenous 21/22-nt-siRdDM loci (group IV) in Col-0 and were co-IPed by 2b protein. Interestingly, the increase in 1C in 2b-bound siRNAs was associated with group IV CHH hypo-DMRs (Figure S3D). The 2b-mediated stabilization of siRNA duplexes might account for the increase in 1C due to antisense siRNAs. A previous study reported that AGO5 predominantly binds siRNAs with 1C^34^, and these results suggested that AGO5 might have a functional role in regulating 21/22-nt-siRdDM. Nevertheless, in DMRs in group IV and V, 2b-coIPed siRNAs are only 21-22-nt (group V) or largely 21-22-nt (group IV) in length, suggesting that DNA methylation of this set of endogenous loci might be guided directly by 21-22-nt siRNAs (e.g., the group V loci), and 21-22-nt siRNAs might also be required and/or act upstream of 24-nt siRNAs-guided RdDM.

In general, body-methylated genes are constitutively expressed at a higher level, and promoter-methylated genes tend to be expressed in a tissue-specific manner^39^. Pol V was found to preferentially associate at regions in which promoters and TEs overlap^40^. RNA-sequence datasets have shown that the active targeting of promoters and TEs by Pol V has functional implications for transcription^40^. We found that the majority of hypo-DMRs in upstream (promoter) regions contain TEs (Figure S6). More than 20% of 2b-coIPed siRNAs were matched with 88.6% of the previously reported potentially Pol V-dependent siRNA loci as well as more than 90% of Pol IV- and Pol IV + Pol V-dependent siRNAs loci^41^ (Figure S7). Transcriptome sequencing of 21/22-nt-siRdDM loci revealed that only a small amount of hypo-DMR loci in both promoter regions and gene bodies displayed a two-fold increase in transcription in the presence of 2b protein (Figure S6). Together with the finding that more than 90% of Pol V-dependent siRNAs are 21-nt^33^, these results suggest a functional role for Pol V in the transcription of 21/22-nt-siRdDM. However, the expression levels of the majority 21/22-nt-siRdDM loci were generally low, and the DNA methylation status at most of them did not impact the expression of these coding genes (Figure S6). Consistent with the previous finding that a high level of DNA methylation had no obvious effect on the regulation of transcription of the two *TAS* genes (*TAS1C* and *TAS3*)^14^, the transcripts of the 5 *TAS* genes in these 21/22-nt-siRdDM loci also displayed no significant changes in 2b-3 plants. Taken together, our data suggest that 21-22-nt siRNAs derived from 21/22-nt-siRdDM loci do not result from changes in transcription and siRNA biogenesis associated with the loss of DNA methylation in the presence of 2b protein, and they further support the notion that 21-22-nt siRNAs play roles in the direct guidance of, as well as alongside 24-nt siRNA in, DNA methylation in a subset of endogenous loci.

The ability of TE to mobilize and insert anywhere in the genome to cause mutations makes them key targets for silencing. Reactivation of TEs causes transposition is previously reported in mutants that have lost both heterochromatic histone modifications and DNA methylation, such as *ddm1*. The *EVD* locus has been shown to have lost methylation and transposition with an increase in copy number in epi15RIL lines derived from the *met1* or *ddm1* background^42^,^43^. We found that TEs with multicopies (either in group IV (*AT2TE18830* and *AT3TE60465*) or group II (*AT1TE68310*)) were likely more susceptible to induced deletions and/or proliferation following 2b-mediated hypomethylation. The inactivation of siRNAs by 2b protein without affecting MET1 or DDM1 may maintain most TEs in an inactive state and retain the low copy numbers.

It has been reported that the accumulation of 21-nt siRNAs from *Athila* TEs prevents transposition and promotes transgenerational TE silencing^44^. It has been suggested that TE reactivation in the vegetative nucleus (VN) may result in the silencing of *Athila* TEs in sperm cells via *Athila* 21-nt siRNAs generated from the reactivated *Athila* TE transcripts from the VN^44^. This model of TE reactivation and potential epigenetic reprogramming is likely to be conserved for reprogramming in the germline in animals to reveal intact TEs in the genome and regulate their activity in gametes. In animals such as *Drosophila*, 25-29-nt PIWI-interacting RNAs (piRNAs) are generated in germline cells and are one means to attack TEs^45^,^46^,^47^. During germline reprogramming, epigenetic marks are first lost and then robustly reset in each generation, resulting in transient, and paradoxical, TE expression^48^,^49^,^50^,^51^,^52^,^53^,^54^,^55^. We found that *Athila TEs* are classified into five hypo-DMR groups (Figure S8). It remains elusive whether the silencing and methylation of 21/22-nt-siRdDM *Athila* TEs in the plant body occurred similarly to pollen and were also mediated by 21/22-nt siRNAs derived from reactivated canonical 24-nt-siRdDM *Athila* TEs. Alternatively, but not mutually exclusive, in the plant body, 21/22-nt-siRdDM TE loci might also be directly targeted by Pol IV, which transcribes hetero-chromatic regions into non-coding transcripts for siRNA generation and feedback reinforcement of DNA methylation and silencing^13^. Nevertheless, the inactivation of siRNAs by 2b protein caused hypomethylation but not necessarily transposition in most of the 21/22-nt-siRdDM TE loci, suggesting that these 21/22-nt-siRdDM TEs might be under rigorous control. Therefore, the loss of siRNAs alone, at least in the plant body, likely does not result in the transposition of TEs with low copy numbers.

Nevertheless, the deletion or proliferation of TEs might result in a decrease and increase in TE copy numbers, leading to the contraction or expansion of TEs in 2b-3 plants. Overexpression of the 2b in plants mainly causes hypomethylation of large amount of genomic sequences that might include genes functions in mediating DNA methylation. We cannot rule out that this might result in enhancing methylation of certain sequences, as about 5000 loci hyper-DMRs were also found in 2b-3 plants. Our recent small RNA deep sequencing and blotting analysis indicate a repetitive DNA origin for CMV satellite RNA in the *Nicotiana* plant genome, a small noncoding subviral RNA pathogen that depends on CMV as a helper virus for its replication and spread in plants^56^. The “cut-off” TE segments are no longer “junk DNA” and could also be the potential origin of the noncoding subviral DNA/RNA pathogen.

In summary, by classifying the various lengths of 2b-coIPed siRNAs in parallel with hypo-DMRs in 2b transgenic plants, we found that, approximately 10% of the 2b-coIPed siRNAs were mapped to the hypo-DMRs. The DNA methylation of a subset of these hypo-DMRs was either directly guided by 21-22-nt siRNAs or required 21-22-nt siRNAs to function upstream of the 24-nt siRNAs-guided RdDM. Similarly to the canonical 24-nt-siRdDM, the majority of the 21/22-nt-siRdDM loci were enriched for CHH and CHG. The expression levels of the 21/22-nt-siRdDM coding genes were generally low, and their DNA methylation status did not impact the expression of these genes. The 2b-mediated hypomethylation of TEs potentially resulted in the deletion and transposition of TEs with multicopies.

## Methods

### Bisulfite Sequencing and Bioinformatic Analyses

Genomic DNA was extracted from *Arabidopsis* leaves via the CTAB method^57^. Library construction and bisulfite sequencing were accomplished by BGI (Table S3). SOAP^58^ was used to align the clean reads against reference genome (Supplemental Methods).

DMRs were identified by comparison of the Col-0 and 2b-3 methylomes using windows that contained at least five cytosines sliding at one cytosine, and CG, CHG and CHH were analyzed separately. A DMR was identified if the P-value from Chi-squared test was ≤0.05.

### Small RNA sequencing and data Analysis

Total RNA was isolated from wide-type Col-0 and 2b-3 plants using hot-phenol extraction^59^. For 2b-associated sRNAs sequencing, aerial tissues from 6myc-SD2b transgenic plants were subjected to immunoprecipitation using anti-α-myc Agarose (Abmart). After washed with IP buffer twice, the immunoprecipitates was incubated in 600 μL Trizol (Invitrogen). After extraction with 0.2 (v/v) chloroform, sRNAs were precipitated with an equal volume of isopropanol.

Small RNAs library construction and Illumina sequencing was performed by BGI (Supplemental Methods). sRNA northern blot was used to verify the miRNA expression (Figure S9).

### sRNAs map to DMRs

The sRNAs that perfect matched to genome were included in our analysis. Blastn was used to search the sRNAs perfect match to DMRs or overlap with DMRs, allowing for both unique and multiple mapping reads. sRNAs density was the RPM normalized by DMRs length. Heatmap and boxplot of sRNAs density were performed by R program.

### Transcriptome Sequencing

Library construction and Illumina sequencing was performed by BGI. All clean reads were aligned against both *Arabidopsis* genomic and cDNA (TAIR10) (Supplemental Methods). The gene expression level is calculated by using RPKM^60^ method (Reads Per kb transcriptome per Million reads). FDR (False Discovery Rate)^61^ were used to correct the diversity test P-value.

### Southern blot

Ten μg of DNA was digested overnight with endonuclease (Thermo Scientific), separated by a 1% agarose gel and transferred to HyBond-NX membrane and cross-linked with UV irradiation. The DNA probes were generated by PCR, and the primers specific for each TEs were listed in the Table S4. The probes were radioactively labelled by [α-^32^P]-dCTP using a Rediprime^TM^ II Random prime Labelling System (Amersham Pharmacia Biotech). Hybridization was performed in hybridization buffer at 65 °C overnight. After washed with 2 × SSC/0.2% SDS twice, the blots were exposed to a storage phosphor screen for 10 h. The hybridizing signals were visualized using a phosphorimager.

## Additional Information

**How to cite this article**: Zhao, J.-H. *et al.* Genome-wide identification of endogenous RNA-directed DNA methylation loci associated with abundant 21-nucleotide siRNAs in *Arabidopsis. Sci. Rep.* **6**, 36247; doi: 10.1038/srep36247 (2016).

**Publisher’s note:** Springer Nature remains neutral with regard to jurisdictional claims in published maps and institutional affiliations.

## Supplementary Material

Supplementary Information
