# Supplementary material for: Genome-wide identification of endogenous RNA-directed DNA methylation loci associated with abundant 21-nucleotide siRNAs in Arabidopsis
Source: Sci Rep. 2016 Oct 27;6:36247. doi: 10.1038/srep36247 (PMC5081565; doi:10.1038/srep36247)
Supplement: Supplementary Information [file srep36247-s1.pdf]

Genome-wide identification of endogenous RNA-directed DNA methylation loci associated with abundant 21-nucleotide siRNAs in *Arabidopsis*

Jian-Hua Zhao[1]*, Yuan-Yuan Fang[1], Cheng-Guo Duan[1#], Rong-Xiang Fang[1], Shou-Wei Ding[2] and Hui-Shan Guo[1]*

[1]State Key Laboratory of Plant Genomics, Institute of Microbiology, Chinese Academy of Sciences, Beijing 100101, China

[2]Department of Plant Pathology and Microbiology, Institute for Integrative Genome Biology, University of California, Riverside, CA 92521, USA

[#]Current address: Department of Horticulture and Landscape Architecture, Purdue University, West Lafayette, IN 47907

*Corresponding author: Hui-Shan Guo

E-mail: guohs@im.ac.cn

Tel: 010-64847989 Fax: 010-64847989

Jian-Hua Zhao

E-mail: zhao_jian_hua@hotmail.com

Tel: 010-64847989 Fax: 010-64847989

Supplemental Methods

Plant growth

Seeds of *Arabidopsis thaliana* accessions Col-0 and 2b transgenic plant 2b-3 were sown on MS medium, imbibed for 2 days at 4 °C, and then moved to green house. After about 10 days, planted the seedlings in soil with 16 h light (cool white light supplemented with incandescent) and 8 h dark at constant temperature of 23 °C.

Leaf tissues of 8 plants from Col-0 and 2b-3 (the 3rd generation F3) were harvested and pooled for DNA, RNA extraction and library construction.

Bisulfite Sequencing and Bioinformatic Analyses

Library construction and bisulfite sequencing were accomplished by BGI (http://www.genomics.cn/en/index). Genomic DNA was sheared by sonication with fragment length in the 100 and 300 bp ranges. Adaptor sequences were ligated to shear end-repaired DNA. The DNA fragments were treated by Bisulfite (ZYMO EZ DNA Methylation-Gold kit). The genomic DNA was treated by sodium bisulfite with a conversion rate higher than 99% (Table S3).

SOAP [1] was used to align the clean reads to reference genome TAIR 10 (soap –v 0 –r 5 –M 0 –a clean.fa –d tair10.fa –o match_genome_reads). Aligned reads cover more than 20 folds of the reference genome and overall unique read coverage of >90% of all cytosines in the genome with at least four reads (Table S3).

The ratio of methylation reads to total reads was used to calculate the average methylation level of reads covered region. Genome-wide coding gene structures are defined by 7 different features to calculate the average methylation level (Figure 1B), which are denoted on the X axis (a-g) [2]. The length of each feature was normalized and divided into equal numbers of bins. Each dot denotes the mean methylation level per bin, and the respective lines denote the 5-bin moving average. Each feature was analyzed separately for each cytosine context [2].

Small RNA sequencing and data Analysis

Small RNAs library construction and Illumina sequencing was performed by BGI (http://www.genomics.cn/en/index). After removal of adaptor sequences, total reads

were mapped perfectly to the *Arabidopsis* genome (TAIR10), and with lengths 18-30-nt were included in our analysis. Normalized reads per million sequences (RPM) represented the expression level of sRNAs. The heatmap and box plot of the siRNA density (Figure 4A, B) were plotted by the function of heatmap() and boxplot() in R program(https://www.r-project.org/) respectively.

Small RNAs blot was used to test the expression of miR156, miR168 and tasi255 (Figure S9). The results show that small RNAs blot results in accordance with small RNAs sequencing data (Figure S9A-D), and 2b protein did not affect the expression of miR156. After total sRNAs reads from Col-0 and 2b-3 libraries were normalized to miR156 which accumulated to a similar level in Col-0 and 2b-3 libraries，21-nt sRNAs increased was observed (Figure S9E).

Transcriptome Sequencing

After having the raw data, FastQC (http://www.bioinformatics.babraham.ac.uk/index.html) was used to quality control on raw reads. SOAP2 [1] was used to perform the alignment. No more than 5 mismatches are allowed in our analysis.

The absolute value of Log2Ratio (⩾1) and FDR [3] (FDR≤0.01) as the threshold to judge the significance of gene expression difference between samples.

Statistic analysis

All the statistic analyses used in this study were calculated by R program. T-test, proportion test, chi-square test, and Mann-Whitney U test were used the function of t.test(), prop.test(), chisq.test(), and wilcox.test() respectively.

Reference

1 Li, R. *et al.* SOAP2: an improved ultrafast tool for short read alignment. *Bioinformatics* **25**, 1966-1967, doi:10.1093/bioinformatics/btp336 (2009).

2 Madhusoodanan, U. K. & Rao, D. N. Diversity of DNA methyltransferases that recognize asymmetric target sequences. *Critical reviews in biochemistry and molecular biology* **45**, 125-145 (2010).

3 Kim, K. I. & van de Wiel, M. A. Effects of dependence in high-dimensional multiple testing problems. *BMC bioinformatics* **9**, 114, doi:10.1186/1471-2105-9-114 (2008).

Table S1. Summary of small RNAs sequencing dataset.

Sample	Total reads	Clean reads	Mapped reads
**Col-0**	19697310	19381517	16144110
**2b-3**	18075032	17709761	13984724
**2b-coIPed**	10424631	7869739	6221062

Table S2. The proportion of DMRs matched small RNAs corresponding to CG, CHG and CHH loci.

		CG	CHG	CHH
**Hypermethylation**	**sRNAs matched loci**	1413	938	1001
	**Total loci**	2729	1031	1333
	**Matched ratio**	52%	91%	75%
**Hypomethylation**	**sRNAs matched loci**	2223	3644	12516
	**Total loci**	4298	4113	15141
	**Matched ratio**	52%	89%	83%

Table S3. Summary of bisulfate sequencing datasets from Col-0 and 2b-3.

sample	Conversion ratio	Raw reads	Genome average cover depth
**Col-0**	99.35%	76,536,140	27.84
**2b-3**	99.41%	63,803,942	22.39

Table S4. The primers for TE southern blot.

TE	Primers（5'-3'）	Restriction Enzymes
AT5TE04360	AAAGGATGACGGTAATAAGGATGATGG<br>ACTTTGGTCGCTCTTCCAAGTGTTT	*SspI*<br>(Thermo Scientific)
AT2TE18830	TTCGGTGTTGGAATGATCTATT<br>TTCAGCCATGTATCTTGGACTA	
AT3TE60465	GCTGGTCGGGAAGTGAATCTGA<br>ATGGAGGCGGTTAGCAAGTTAG	
AT1TE68310	GAGGTGGTTTCATATTTGTTTG<br>TTGAGTTATTAGAAATCGTCCC	*Hind III*<br>(Thermo Scientific)
AT1TE46265	GCTAAGAAACTGGGCTTCAACA<br>GAGGCAACATTTAACTTCCTATACTC	
AT3TE67540	AAAACAATTCACTGCCTTCTAC<br>GACTCTGCTGTGGCTGCTAAAC	
AT3TE48455	CACCCTCTTGGATAATGGACAC<br>AAACCTCAACGCCCTTGCTACA	

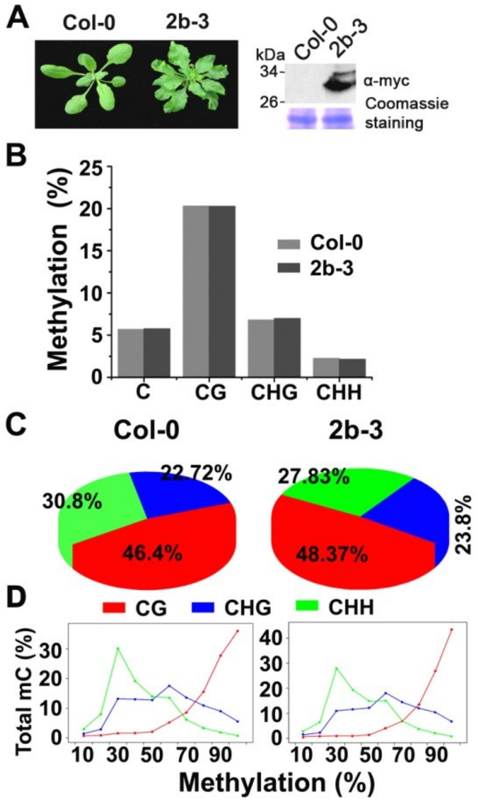

Figure S1. Global trends in DNA methylation in Col-0 and 2b-transgenic line 2b-3.

(A). The phenotype of 2b-transgenic line 2b-3 and the accumulation of the 2b protein in 2b-3 plants. (B). Bisulfite sequencing data showing the percentages of total C, CG, CHG, and CHH methylation genome-wide in Col-0 and 2b-3. (C). The fraction of methylcytosines identified in each sequence context for Col-0 and 2b-3. (D). The distribution of the percentage of methylation in each sequence context. The y-axis indicates the fraction of the total methylcytosines displaying each percentage of methylation (x axis), in which the percentage of methylation was determined as the fraction of reads at a reference cytosine containing cytosines following bisulfite conversion. The fractions were calculated within bins of 10%, as indicated on the x-axis.

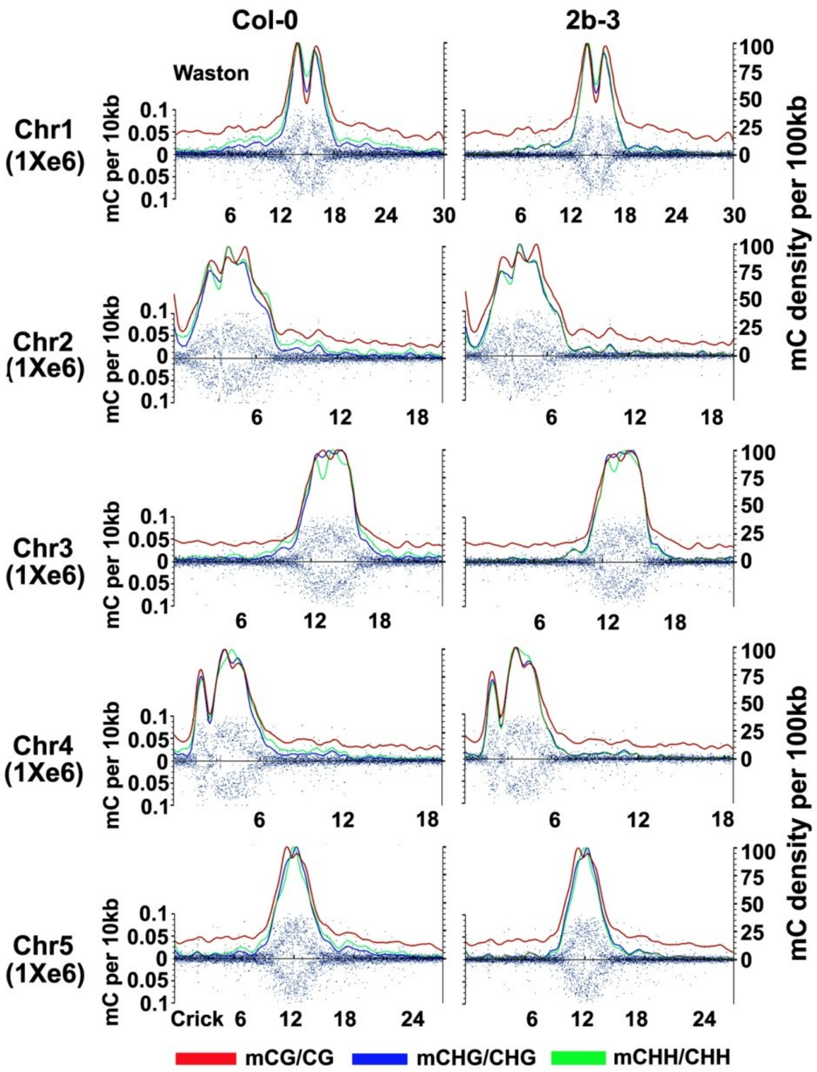

Figure S2. Methylcytosine density throughout each chromosome in Col-0 and 2b-3. Dots denote the methylcytosine density in Col-0 in 10-kb windows throughout each chromosome. Smooth lines represent the methylcytosine density in CG, CHG and CHH contexts in Col-0, and they were counted in 100-kb bins.

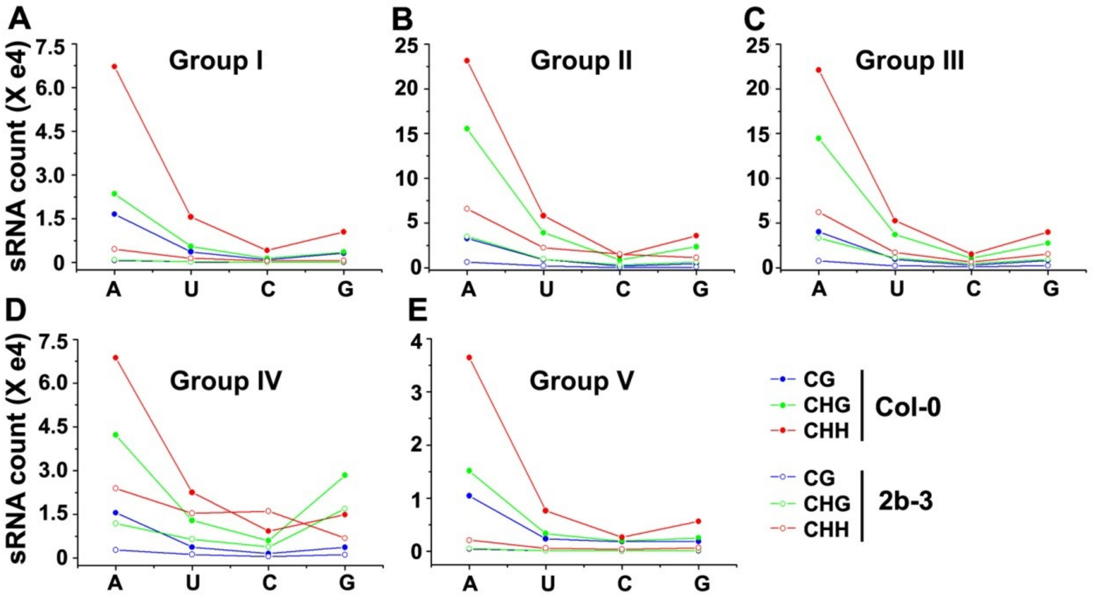

Figure S3. The numbers of each nucleotide at the 5'-terminus of siRNAs that matched with each Hypo-DMR group.

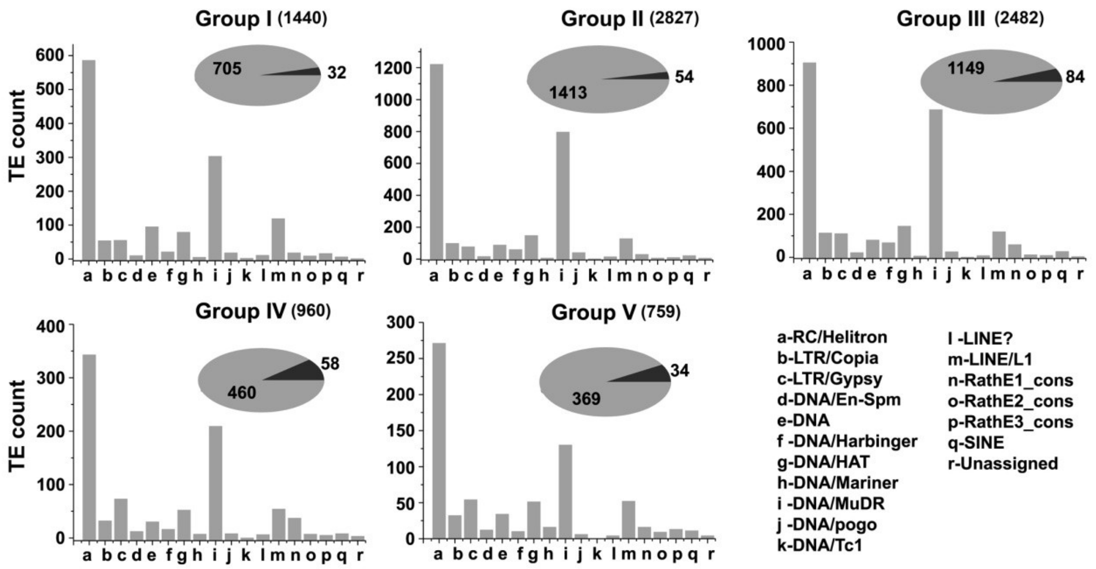

Figure S4. Distribution of TEs in hypo-DMRs in each superfamily.

Column diagrams showing the numbers of TEs in each hypo-DMR group in each of 18 TE superfamilies. The total numbers of TEs in 15 subfamilies have been analyzed previously[12] in each group and are indicated in the gray oval and dark gray portion.

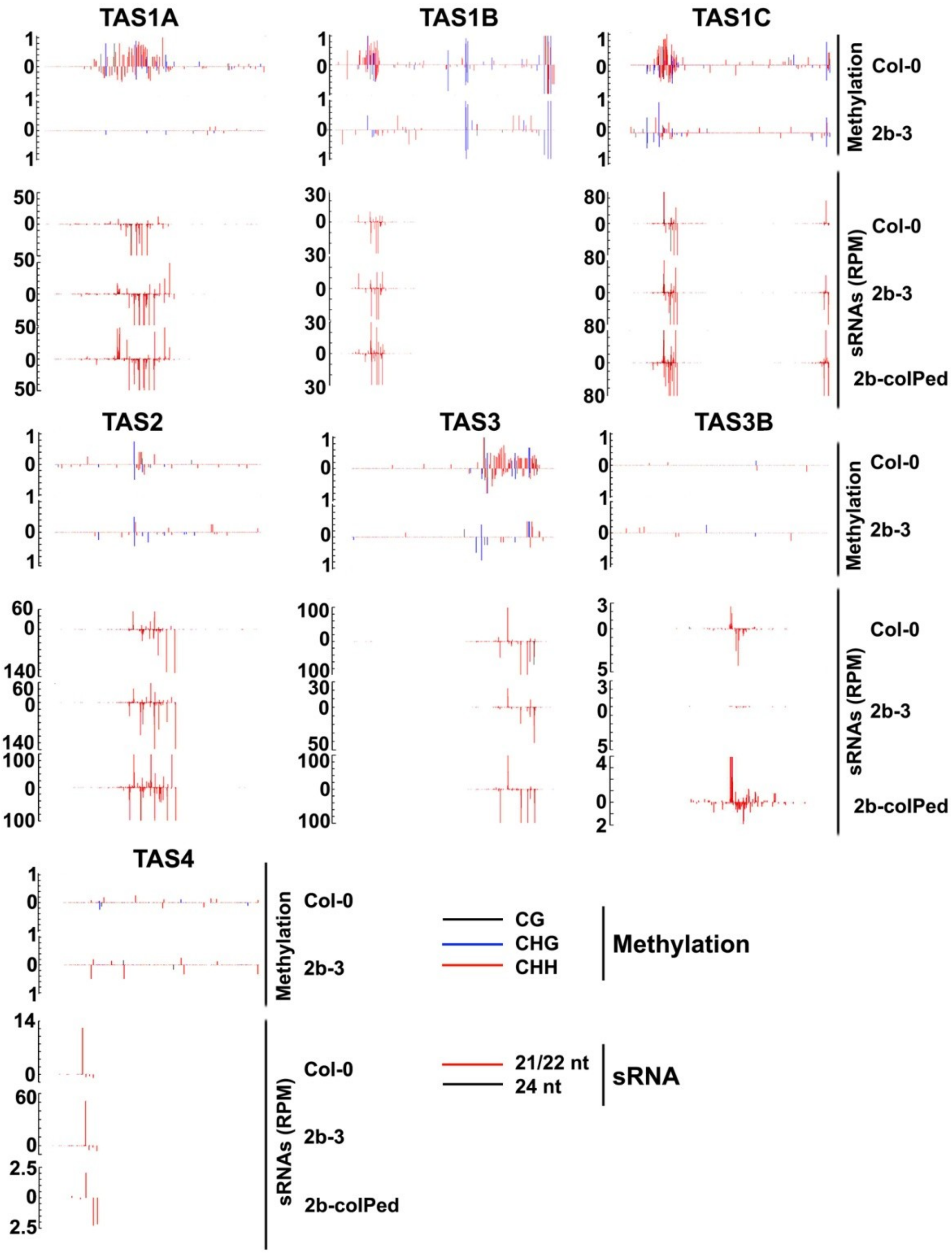

Figure S5. DNA methylation profiles of *TAS* loci and distribution of *TAS*-derived siRNAs.

DNA methylation status of *TAS* gene bodies in Col-0 and 2b-3 and distribution of *TAS*-derived siRNAs in Col-0 and 2b-3 plants and 2b-coIPed *TAS*-derived siRNAs. The vertical lines show the methylation level of each cytosine context and different siRNA lengths.

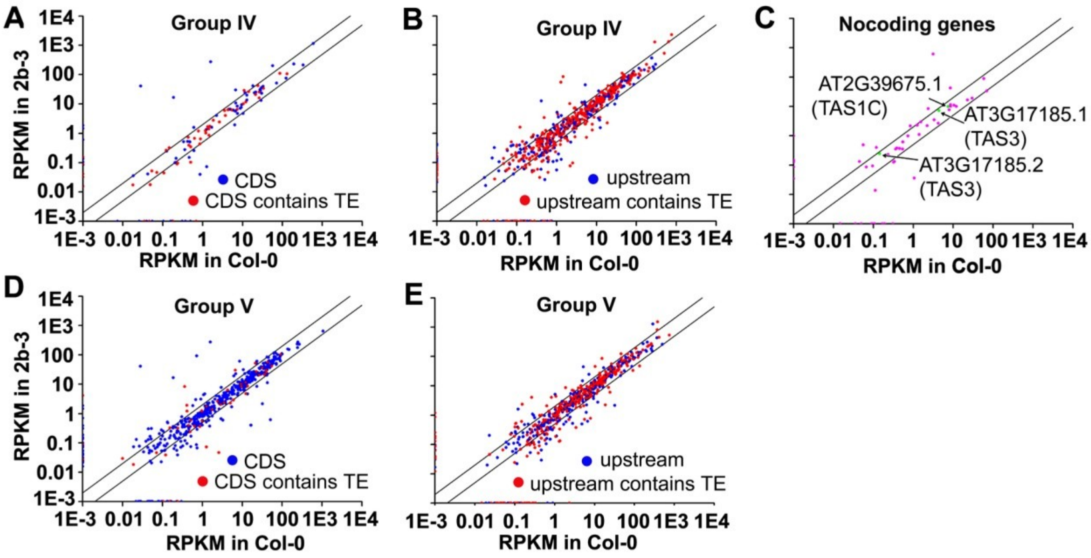

Figure S6. Transcription levels of the coding genes in hypo-DMR group IV and V in Col-0 and 2b transgenic plants.

The expression levels of coding genes for hypo-DMRs identified in gene bodies (CDSs) (A, D), in upstream regions (B, E), or in *TAS* genes (C). Red and blue dots represent the CDSs or upstream regions with or without overlap with TEs. The x-axis shows the gene RPKM in Col-0, and the y-axis shows the gene RPKM in 2b-3. The black lines show the threshold for the 2-fold change in RPKM. 11 of 172 genes (A), 27 of 665 genes (B), 26 of 592 genes (D), and 30 of 601 genes (E) displayed a significant increase ($P < 0.05$, $FDR < 0.001$).

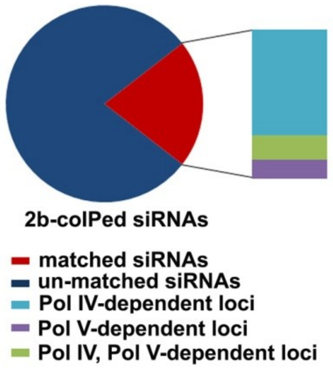

Figure S7. Comparison of 2b-coIPed siRNAs with Pol V-dependent siRNA loci

Over 20% of the 2b-coIPed siRNAs were matched with 88.6% of the previously reported potential Pol V-dependent siRNA loci, 99.5 % of the Pol IV-dependent siRNA loci and 91.2% of the Pol IV, Pol V-dependent siRNA loci [41].

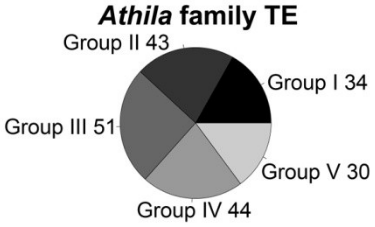

Figure S8. The number of *Athila* family TEs in the five hypo-DMR groups.

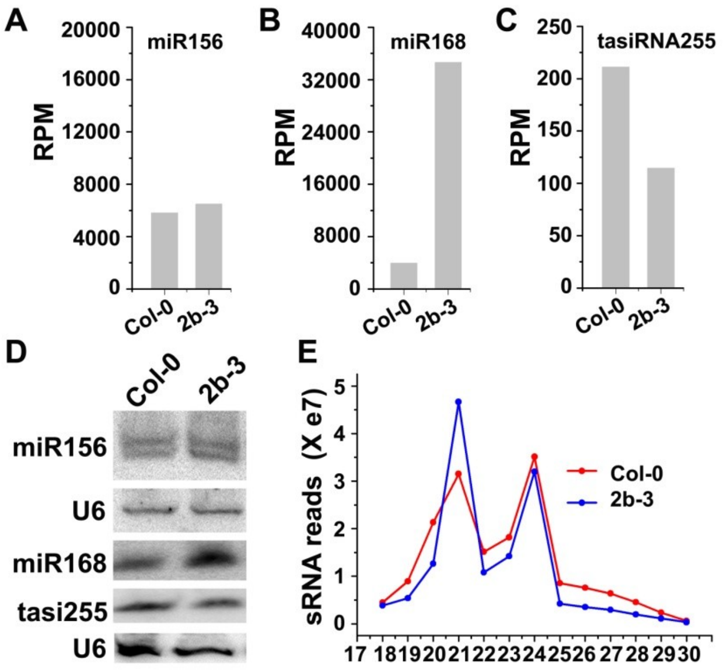

Figure S9. Analysis of siRNA deep sequencing data

(A. B. C). The RPM of miR156, miR168 and tasiRNA255 in Col-0 and 2b-3 siRNAs libraries. (D). RNA blotting analysis of the accumulation of miR156, miR168 and tasiRNA255 in Col-0 and 2b-3. (E). The total siRNA reads from the Col-0 and 2b-3 libraries were normalized to miR156, which accumulated to a similar level in Col-0 and 2b-3, as shown in (A) and (D).
